# Online Machine Learning for Accelerating Molecular Dynamics Modeling of Cells

**DOI:** 10.3389/fmolb.2021.812248

**Published:** 2022-01-27

**Authors:** Ziji Zhang, Peng Zhang, Changnian Han, Guojing Cong, Chih-Chieh Yang, Yuefan Deng

**Affiliations:** ^1^ Department of Applied Mathematics and Statistics, Stony Brook University, Stony Brook, NY, United States; ^2^ Oak Ridge National Laboratory, Oak Ridge, TN, United States; ^3^ IBM Thomas J. Watson Research Center, Yorktown Heights, NY, United States; ^4^ Mathematics, Division of Science, New York University Abu Dhabi, Abu Dhabi, United Arab Emirates

**Keywords:** online machine learning, molecular dynamics, computational fluid dynamics, equation of motion, multiscale modeling

## Abstract

We developed a biomechanics-informed online learning framework to learn the dynamics with ground truth generated with multiscale modeling simulation. It was built on Summit-like supercomputers, which were also used to benchmark and validate our framework on one physiologically significant modeling of deformable biological cells. We generalized the century-old equation of Jeffery orbits to a new equation of motion with additional parameters to account for the flow conditions and the cell deformability. Using simulation data at particle-based resolutions for flowing cells and the learned parameters from our framework, we validated the new equation by the motions, mostly rotations, of a human platelet in shear blood flow at various shear stresses and platelet deformability. Our online framework, which surrogates redundant computations in the conventional multiscale modeling by solutions of our learned equation, accelerates the conventional modeling by three orders of magnitude without visible loss of accuracy.

## Introduction

Molecular dynamics (MD) is a computer simulation method that analyzes how atoms and molecules move and interact during a fixed period of time (Jia et al., 2020). It is critical for novel knowledge discovery and solution design but due to their extremely high computational cost these simulations often run at a limited scale. With the aid of today’s high-performance computing (HPC) systems, MD simulations are accelerated, however, complex biological processes in computational biomedicine and bioengineering still require a massive computing time of up to years. One of the most time-consuming procedures in MD simulation is the calculation of physics governing equations of motion. The structures of these governing equations are often previously known and studied with physics first principles, but the determination of these equation parameters is what MD is truly used for. One such demonstration is the motion of isolated ellipsoids immersed in a steady viscous shear flow, described by the Jeffery orbits equation (JOE) (Jeffery, 1922) introduced in 1922, which is widely used as a benchmark to parameterize and validate the numerical models of cells in biofluids. The JOE demonstrates that flowing objects tumble with many infinite marginally stable periodic orbits, which vary sensitively by flow conditions or object shapes.

Sparse, incomplete, noisy, or heterogeneous data pose a natural challenge to modeling biological, biomedical, and behavioral systems. In principle, direct numerical simulations can fill this gap and generate the missing data. However, simulations themselves can be limited due to poorly calibrated parameters. There is, therefore, a pressing need to develop robust inverse methods that are capable of handling sparse data (Peng et al., 2020). Machine learning (ML), an increasingly promising technology for knowledgebase discovery in the biological and biomedical sciences, among many others (Kissas et al., 2020; Raissi et al., 2020), tends to mispredict or fail due to lack of conceptual consideration or mishandling of noisy and sparse data (Peng et al., 2020). This could be rectified with science-informed prior augments the ML technology. In essence, physics-based numerical modeling of complex biological systems is a fundamental tool for gathering ground truth (Teichert et al., 2019). Therefore, the design of our ML, tailored for the applications, determines the physiologically meaningful parameters. The learning framework, informed by biomechanics knowledge, is explainable and conveniently generalizable while it can correct mispredictions of the “black-box” approaches. In this work, we chose to utilize HPC platforms to generate the massive data needed for our ML to learn the parameters for the underlying governing equations.

The integration of HPC and ML poses another challenge in that it demands massive storage capacity and I/O for modeling complex biological systems that rely on a high-dimensional parametric input space. This is particularly acute when creating personalized models with complex geometries and multiple spatial and temporal scales (Peng et al., 2020). Alternatively, a real-time framework that analyzes simulation streaming data on the fly, alleviating most of the burden, motivates us to explore online learning (OL) methods (Agarwal et al., 2008; Wigley et al., 2016) in determining physics governing equations and modeling biological cells in MD simulations. Several practical issues must be addressed before applying OL to learning HPC simulations:

a) Definitions of online optimization objective function to determine the physics governing equations.
b) Management of the online learning and inferencing data.
c) Optimal time to surrogate the online learning in the ongoing underlying simulations.

We address these issues in the design of our biomechanics-informed online learning framework (BIOL). Using multiscale modeling (MSM) to provide the ground truth, we propose a learned Jeffery orbits equation (L-JOE) based on the highly celebrated theory (Jeffery, 1922) and BIOL to study the motion of the object, the fluid, and their interactions. In this application, the equation of motion of platelet in the shear blood flow is fully established by the joint using the L-JOE and BIOL. Such an accurate description of the platelet motion, combined with its advantages in speeding up the MSM of moving cells, may broaden our simulations to other physiological analyses of cells and even clinical medicine.

The holistic approach of BIOL and L-JOE, that we developed for the exemplary application with the potential of generalization to other problems, enabled us to minimize the redundant time-consuming calculations in conventional multiscale simulation. More specifically, our major contributions are as follows.

a) We modeled the motion and metamorphosis of platelets in biofluids, a primary apparatus for examining the rheology of these biomedical phenomena, which resulted in high-fidelity dynamics simulation data for biomedical discoveries.
b) We proposed the first online learning-based framework for deriving the parameters for governing equations and further accelerating simulations by correlating with high-fidelity *in silico* data.
c) We speed up the processing time from months to hours for the online analysis of streaming simulation data of terabytes data files.
d) We reclaimed more detailed physics, omitted in JOE, to enable adaptive determination of equation parameters varying with, *e.g.*, fluid conditions, which enables numerical simulations of complex biomedical problems.

The remainder of our manuscript will address the following: *Related Work* introduces the background study and related work. *Methods* states the problem along with our proposed L-JOE and BIOL for the equation’s structure and parameters respectively. *Application to Platelets in Shear Blood* applies our methods to platelets in shear blood. We explain the MSM simulating system for generating ground truth and present the implementation details of our integrated systems. *Results Analysis* analyzes the performance of our methods for describing the dynamics of platelets in shear blood flow. *Discussion* discusses the broader impact of our methods, summarizes the observations on L-JOE and BIOL then outlines future work.

## Related Work

Modeling of the motion and metamorphosis of cells is becoming a primary apparatus for examining the rheology of these biomedical phenomena. Such study has come a long way, for example, Einstein (1911) calculated an effective viscosity for a dilute suspension of noncolloidal hard spheres and showed that the effective viscosity of the suspension increases linearly with the volume fraction of spheres. Understanding the dynamics of even a single object in shear is important to determine the rheology of the suspension of anisotropic objects. The dynamics of irregular objects such as axisymmetric ellipsoids are significantly more intricate than that of spheroids under shear (Edwardes, 1892; Jeffery, 1922; Chwang and Wu, 1974). At Reynolds number zero, spheroids and long slender bodies in shear flow undergo a periodic motion. A century ago, Jeffery (1922) studied the motion of a neutrally buoyant ellipsoid of revolution in a simple uniform shear flow in the absence of inertial and Brownian forces, known as JOE which is widely used as a benchmark to parameterize and validate numerical models of cells in biofluids. The study found that the ellipsoid’s axis of revolution rotates on infinitely many degenerate periodic Jeffery orbits. JOE solution is degenerate in the sense that the late time orientation of the ellipsoid depends on its initial orientation. Recently, Einarsson et al*.* (Byron et al., 2015; Candelier et al., 2015; Einarsson et al., 2015a; Einarsson et al., 2015b; Rosén et al., 2015; Einarsson et al., 2016) showed theoretically that, in the limit of weak flow and inertia, the degeneracy of Jeffery orbits is indeed lifted. Much of the work on the motion of objects in complex fluids has focused on the effects of flow conditions, according to a recent review (Shaqfeh, 2019) that summarizes the advances in the rheology of the suspensions of objects in viscoelastic fluids. However, they seem to be too idealistic to address applications involving shape-varying objects and their interactions with the flow.

Efforts have been made (Zhang P. et al., 2014; Zhang et al., 2017; Gupta et al., 2019) to adapt the JOE for various applications where the flow conditions or object shapes change dynamically. Zhang P. et al. (2014) compared the MSM data with the JOE solution for a flowing oblate-shaped platelet in Couette flows for rigid and deformable platelet models in which the rotation angle, its velocities, and its acceleration versus the total strain were analyzed. The flipping trajectories generated by MSM for a rigid platelet in Couette flow resemble remarkably well the JOE solution and, for the motion of deformable platelets in a more realistic and physiological setting, such favorable potential of JOE quickly diminishes as expected. A three-way comparison of the JOE solutions, MSM data, and the *in vitro* measurements revealed that the latter two agree with each other and both differ from the former because JOE neglected the fluid-platelet interactions and platelet surface quivering or bigger shape changes. The clear path forward is to ameliorate JOE for a more accurate representation of the object and flow while taking advantage of its vast saving in computing. A straightforward solution by Einarsson et al*.* (Einarsson et al., 2015a; Einarsson et al., 2015b; Byron et al., 2015; Candelier et al., 2015; Rosén et al., 2015; Einarsson et al., 2016) derived an equation of motion for a neutrally buoyant ellipsoid in steady shear, but the direct computation of the object inertia that affects the fluid mutually is a daunting task. In our manuscript, the dynamics of a neutrally buoyant oblate in the shear flow of a viscous fluid are studied. The oblate is so sized that it is too small to experience inertial forces and too big to have Brownian motion. The learned Jeffery orbits observed in our study indeed deviate while their degeneracy remains due to the symmetry of the constitutive equations.

The use of simulated high-fidelity data as ML objects has received attention, which has advanced detailed deterministic models and their coupling across scales (Deist et al., 2019; Li et al., 2020; Raissi et al., 2020). Raissi et al*.* (Raissi et al., 2020) developed this approach to quantify fluid flow and extract velocity together with pressure fields. Their method exploits knowledge of Navier-Stokes equations, which govern the dynamics of fluid flow in many scientifically relevant situations, illustrated by examples such as blood flow in an aneurysm. Yazdani et al. (2020) developed a new systems-biology-informed deep learning algorithm that incorporates the system of ordinary differential equations into the neural networks. However, problems may arise when dealing with sparse, biased, or time-dependent data, in which cases the naive use of machine learning can result in ill-posed problems and generate non-physical predictions (Peng et al., 2020). The existing online learning techniques implemented on HPC (Tuncer et al., 2018; Borghesi et al., 2019; Netti et al., 2019) fail to integrate underlying physics prior which constrains the space of admissible solutions. Therefore, there are still challenges for achieving honest precision across the entire scales for general physics processes, but our BIOL opens the door to a new era of real-time analysis for *in silico* simulations that could save significant computing time and disk space while extending the reach of physics searches and precision measurements at the biological processes and beyond.

## Methods

Our BIOL, designed to be a general purpose for predicting the parameters of the empirical equations, is applied to examine our L-JOE for the motion of oblate-shape cells in biofluids. In BIOL, the big streaming data gathered through MSM experiments provide the ground truth and, conversely, the online learning predictions can feedback with signal and guide the MSM experiments on the fly.

### Learned Jeffery Orbits Equations

**FIGURE 1:**
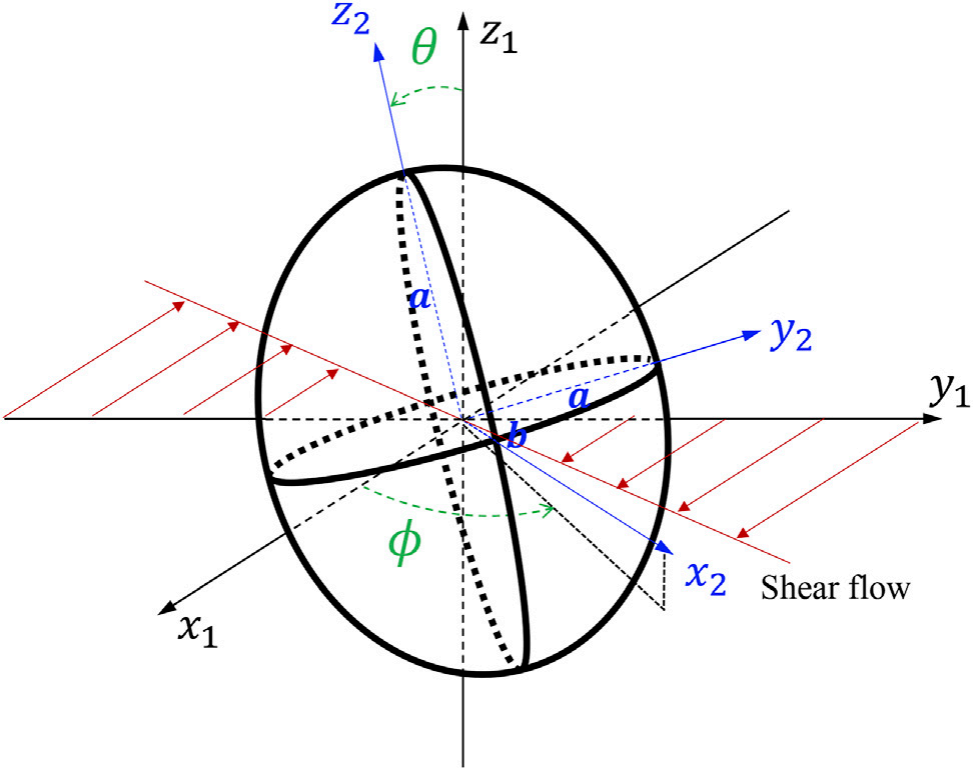
A tumbling oblate with $\left(x_{1},y_{1},z_{1}\right)$ and $\left(x_{2},y_{2},z_{2}\right)$ frames of reference.

We consider an oblate with a given axis of symmetry, and the major (minor) axis a ( b ) with an aspect ratio e=a/b>1 . The oblate is immersed in a laminar flow and is methodically positioned in the flow to eliminate sliding (Figure 1). In JOE (Jeffery, 1922), the rotation angle ϕ and angular speed $\dot{\phi }$ are defined as

$$\phi \left(\dot{\gamma }t\right)=\tan^{-1}\left(\frac{1}{e}\tan\frac{e\dot{\gamma }t}{e^{2}+1}\right)$$

$$\dot{\phi }\left(\dot{\gamma }t\right)=\frac{1}{2}\left(\Lambda \cos\left(2\phi \right)+1\right)$$

where $\Lambda =\left(e^{2}-1\right)/\left(e^{2}+1\right)=\left(a^{2}-b^{2}\right)/\left(a^{2}+b^{2}\right)$ is a geometrical constant measuring the ellipsoidal extent, $\dot{\gamma }$ is the flow shear stress and $\dot{\gamma }t$ is the dimensionless time. Einarsson et al. (2015a) derived the modified Jeffery orbits equation, M-JOE $\left(\phi ,\dot{\phi };\beta_{1},\beta_{2}\right)$ with two correction terms to account for the weak but nonnegligible inertial effects for the motion of a neutrally buoyant spheroid in a steady shear flow

$$\dot{\phi }\left(\dot{\gamma }t\right)=\frac{1}{2}\left(\Lambda \cos\left(2\phi \right)+1\right)+\beta_{1}\sin\left(2\phi \right)+\beta_{2}\sin\left(4\phi \right)$$

where $\beta_{1}$ and $\beta_{2}$ are correction constants. To further improve M-JOE, we propose a learned Jeffery orbits equation, L-JOE $\left(\phi ,\dot{\phi };\kappa_{0},\lambda_{0},\lambda_{1}\right)$ , to incorporate more physics neglected in JOE and M-JOE by introducing three physically meaningful but dimensionless parameters $\left\{\kappa_{0},\lambda_{0},\lambda_{1}\right\}$ ,

$$\begin{array}{l} \dot{\phi }\left(\dot{\gamma }t\right)=\frac{1}{2}\left(\Lambda \cos\left(2\phi \right)+1+\kappa_{0}\right)\left(1+\lambda_{0}+\lambda_{1}\sin\left(2\phi \right)\right)\\ =\frac{1}{2}\left(\Lambda \cos\left(2\phi \right)+1\right)+\frac{1}{2}\lambda_{1}\left(1+\kappa_{0}\right)\sin\left(2\phi \right)+\frac{1}{2}\lambda_{0}\Lambda \cos\left(2\phi \right)+\frac{1}{4}\lambda_{1}\Lambda \sin\left(4\phi \right)+\frac{1}{2}\left(\lambda_{0}+\kappa_{0}+\lambda_{0}\kappa_{0}\right) \end{array}$$

where $\kappa_{0}$ is the fluid-object coupling constant, $\lambda_{0}$ accounts for the coarse grained and $\lambda_{1}$ for the fine-grained deviations from a perfect oblate. When $\kappa_{0}=\lambda_{0}=\lambda_{1}=0$ , our L-JOE truncates to JOE, and when $\kappa_{0}=\lambda_{0}=0,$ $\beta_{1}=\frac{1}{2}\lambda_{1},\beta_{2}=\frac{1}{4}\lambda_{1}\Lambda$ , the correction terms in (Einarsson et al., 2015b) are contained in L-JOE. L-JOE requires only one correction parameter $\lambda_{1}$ to function as well as M-JOE that requires two correlated parameters. The two correction terms in L-JOE, $\frac{1}{2}\lambda_{0}\Lambda \cos2\phi$ and $\frac{1}{2}\left(\lambda_{0}+\kappa_{0}+\lambda_{0}\kappa_{0}\right)$ , describe the oblate deformation and its interaction with surrounding flow.

### Feature Extracting

**FIGURE 2:**
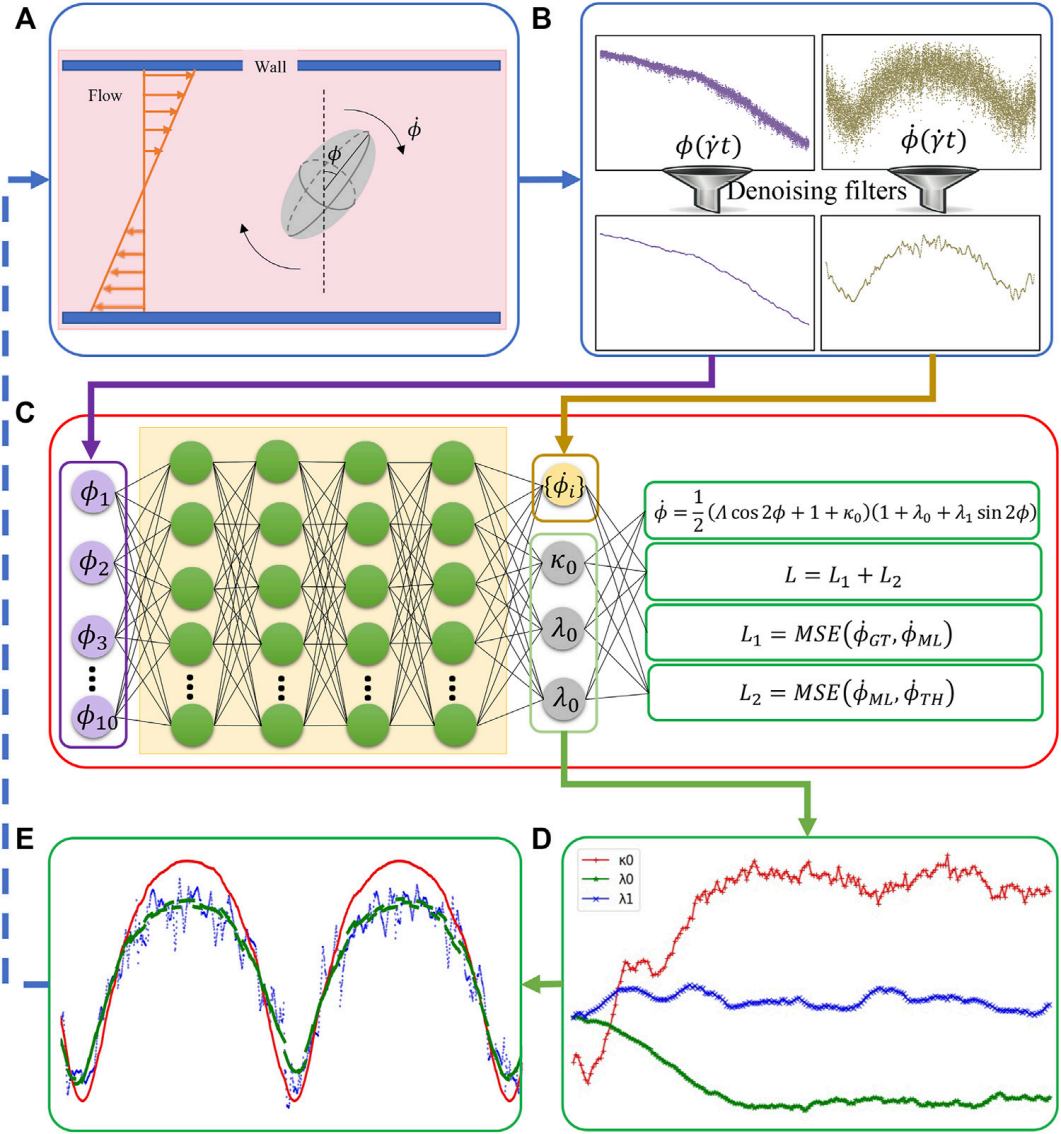
The integration of BIOL with MSM **(A)**. Raw data are pre-processed **(B)** and then sampled for online training **(C)**. The learned parameters **(D)** allow predictions for angular speed **(E)** and comparison of the ground truth (blue), JOE (red), and L-JOE (green) solutions. The L-JOE theory in the right side of panel C describes the relationship between ϕ and ${\dot{\phi }}_{TH}$ .

ML, originating from statistics and computer science, extracts the relationships of data and develops algorithms to process data with the full awareness of domain-specific data (Peng et al., 2020). The BIOL (Figure 2) interprets and selects features guided by biomechanics prior including the understanding of the system. In MSM, the cell motion is described by

$$E_{k}=E_{tr}+E_{ro}=\frac{1}{2}M{\left\|V_{com}\right\|}^{2}+\frac{1}{2}\mathrm{\omega I}\omega^{T}$$

where M is the oblate mass, $V_{com}$ is the center-of-mass (COM) velocity, $\omega =\left(\omega_{x},\;\omega_{y},\;\omega_{z}\right)$ is the angular velocity, and I is the moment of inertia of the oblate. Among the many features, we study the rotation of the oblate in the x-y plane.

### Online Sampling

The input sampling policy (Algorithm 1) requires BIOL to capture the global trends of the time series from the short-term memories without weighty dependence on the latest changes, *i.e.*, the BIOL can capture long-term trends via the online ML that overcomes the pitfalls of short time series. A trivial sampling is to mix randomly selected history data (cold) with the recent data (hot) at a pre-fixed ratio which is highly impractical due to unsatisfiable storage requirements and highly insensitive to online parameter changes. Our more sophisticated policy is to use a probability distribution P(t) , governed by the specific flow conditions, to determine the conformation of cold and hot data reflecting the time such data last became available. At the current time τ , the training dataset is sampled from the most recent time series of window size W following $\int_{\tau -W}^{\tau }P\left(t\right)dt=1$ with [τ−W,τ−w] for cold and [τ−w,τ] for hot, where 0≤w≤W . Each sample represents a short time series, randomly selected based on P(t) . This kind of short time series, containing limited but important short-term information, is used as the element training sample to feed into our neural network.

**ALGORITHM 1:**
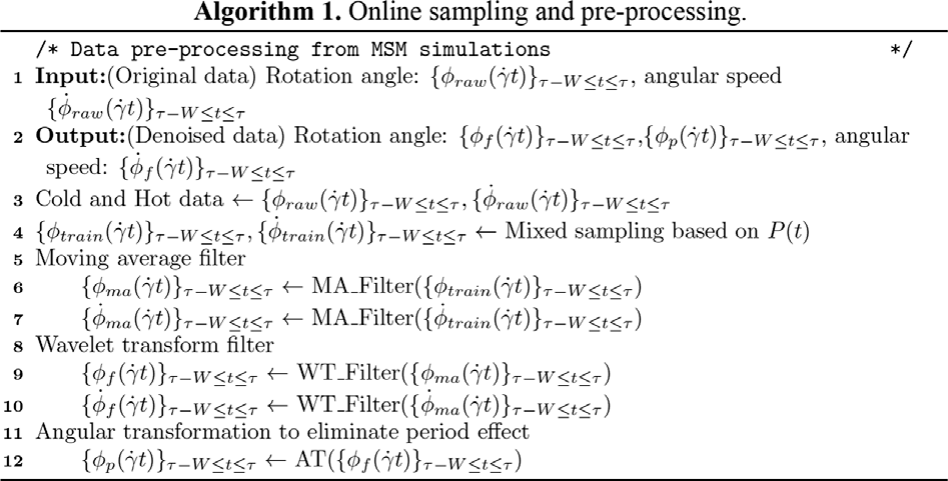


### Data Processing

The preprocessing and denoising filters (Algorithm 1) capture the biomechanics of the cell from the raw MSM data. During this stage, a moving averaging (MA) filter with a fixed size of window and stride, followed by a noise-reducing wavelet transformation (WT) filter. The input data are transformed into low- and high-frequency components as (Zimoń et al., 2016)

$$s\left(t\right)≅\sum_{k\in \mathbb{Z}}A_{J,k}\varphi_{J,k}\left(t\right)+\sum_{j,k\in \mathbb{Z}}D_{j,k}\psi_{j,k}\left(t\right)$$

where

$$A_{J,k}=\int s\left(t\right)\varphi_{J,k}\left(t\right)dt,\varphi_{J,k}\left(t\right)=2^{-\frac{J}{2}}\varphi \left(\frac{t-2^{J}k}{2^{J}}\right),$$

$$D_{j,k}=\int s\left(t\right)\psi_{j,k}\left(t\right)dt,\psi_{j,k}\left(t\right)=2^{-\frac{j}{2}}\psi \left(\frac{t-2^{j}k}{2^{j}}\right).$$

The $A_{J,k}$ and $D_{j,k}$ are the coefficients for the orthogonal relationship between wavelet at resolution level j and input data s(t) where 0<j≤J , $0\leq k<2^{j}$ , and J is the maximum level. The function $\varphi_{J,k}\left(t\right)$ is the smooth approximation after translating and dilating a scaling function φ , and the fine-scale details are generated by an orthonormal basis function $\psi_{j,k}$ , scaling of a mother wavelet ψ . A smoothing estimated signal is reconstructed as inverting the transform by cleaning coefficients with resolution-dependent thresholds. The soft threshold (Donoho and Johnstone, 1994) is employed following

$$\eta_{s}\left(D_{j,k},\mu_{j}\right)=\left[D_{j,k}-\mu_{j}\cdot \mathrm{sgn}\left(D_{j,k}\right)\right]I\left\{\left|D_{j,k}\right|>\mu_{j}\right\}$$ (1)

where I and sgn are indicator and sign functions respectively and $\mu_{j}$ is the threshold at resolution level j .

For rotation angle ϕ , we eliminate the undesired effect of its periodicity by an angular transformation (AT) from $\phi_{f}$ to $\phi_{p}$ with normalization (Algorithm 1)

$$\phi_{p}=\{\begin{array}{cc} 1-\frac{2\phi_{f}}{\pi }, & 0\leq \phi_{f}<\frac{\pi }{2}\\ 1+\frac{2\phi_{f}}{\pi }, & -\frac{\pi }{2}\leq \phi_{f}<0 \end{array}$$

The ground truths may contain numerical errors and the deformability in our simulations involving deformable bodies contaminates measuring the angle and angular speeds, necessitating rectifications of the rotation angle by online learning for a parameter, L-JOE $\left(\phi -\phi_{0},\dot{\phi }\right)$ , to mitigate the prediction errors of the physics parameters.

### Online Training and Inferencing

A customized loss function, measuring the difference between data from MSM and basic physics, is introduced for predicting parameters in BIOL (Algorithm 2). The two terms in the loss function measure the coupled effects from both MSM (the first term) and basic physics (the second term).

$$L\left({\dot{\phi }}_{GT},{\dot{\phi }}_{ML},{\dot{\phi }}_{TH}\right)=L_{1}\left({\dot{\phi }}_{GT},{\dot{\phi }}_{ML}\right)+L_{2}\left({\dot{\phi }}_{ML},{\dot{\phi }}_{TH}\right)$$

where the first term trains $N\left(\overset{↔}{W},\overset{↔}{b}\right)$ to optimize the weights and the biases $\overset{↔}{W},\overset{↔}{b}$ while the second term trains the proposed equation to optimize its parameters. Naturally, a function $L_{3}\left({\dot{\phi }}_{GT},{\dot{\phi }}_{TH}\right)$ , the difference between the ground truth and the theory, evaluates the predictions. $L_{1},L_{2},\;L_{3}$ are norm functions where we generally use Mean Squared Error (MSE). The training and validation use the most recent randomly sampled historical data and evaluation uses the freshly generated data for accuracy measurements, to accommodate the fact that dynamics are highly time sensitive. The last component of BIOL is to correlate ML with MSM and integrate them with the time series for online learning, inferencing, and feedback with signal ζ. Coupled ML and MSM leverage their respective strengths to identify the system equations and parameters of the ill-posted problems with sparse and noisy data at multiple spatial and temporal scales (Peng et al., 2020).

**ALGORITHM 2:**
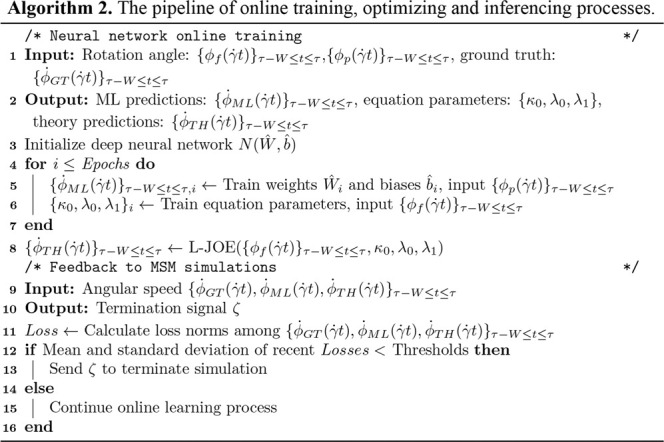


## Application to Platelets in Shear Blood

We use MSM to gather the ground truth for the motion of deformable platelets in shear blood flows at the nano-to-micron spatial, with the associated temporal, scales. The MSM of platelet motion in the blood, while considering inner degrees of freedom of the platelet as well as the flows at multiple spatial and temporal scales, is extremely time-consuming, even though MSM is the method of choice for studying large and complex systems in biology, material science, and fluid dynamics. The dynamics of platelets in the blood plays an important role in the formation of thrombosis, a common pathology underlying cardiovascular diseases, which accounts for over 30% of all deaths globally (Casa et al., 2015; Benjamin et al., 2019). It also plays a potential role in triggering deaths from COVID-19 infection (Koupenova, 2020).

### Multiscale Models for Deformable Platelets

A microchannel is simulated by a rectangular tube of 16 × 16 × 8 $\mu m^{3}$ with periodic boundary conditions in x - and z -dimensions (Gupta et al., 2019). The blood vessel walls are modeled as the top and bottom y-boundaries with no-slip boundary conditions. The entire system consists of 1,091,360 fluid particles and 140,303 platelet particles of which 40,446 and 32,853 and 67,004 atoms are for the cytoskeleton, the cytoplasm, and membrane respectively (Zhang P. et al., 2014; Zhang et al., 2017; Gupta et al., 2019). The platelet model is depicted in (Zhang et al., 2017) in which a quiescent platelet is modeled as a discoid-shaped spheroid with a 2 µm semi-major axis and 0.5 µm semi-minor axis. The peripheral zone is modeled as a homogeneous elastic material bilayer constructed by the 2D triangulation method. A shell of 300 Å thickness represents the phospholipid bilayer deformable membrane (100 Å) and an exterior coat (150–200 Å). The organelle zone, represented by the cytoplasm, is composed of homogenous nonbonded particles filling the gap between the membrane and the cytoskeleton. At the fluid-platelet interface, the membrane prevents fluid particles from penetrating while maintaining the fluid-platelet interactions (Zhang N. et al., 2014). The cytoplasm rheology and its resultant deformability are modeled with a viscosity ranging from 4.1 to 23.9 mPa s using a Morse potential (Bluestein et al., 2013). The cytoskeleton consists of two types of actin-based filaments: a rigid filamentous core and an assembly of radially spanning elastic actin filaments that mediates the contractility. A carbon-70 structure is used to generate the oval shaped core. Each actin filament, individually extensible, is tethered to the core. An *α*-helical structure, mimicking the spring-loaded molecular mechanism, can stretch its spiral conformation continuously. The shear blood flow is modeled by a counter Couette flow by sliding two opposing walls in x -direction to emulate the flow shear stresses of {50, 100, 200, 300} in dyne/cm^2^ and we use the same force field as Gupta et al. (2019). The integration timestep size is 416 ps with states collected every 40ns in our modified LAMMPS (Plimpton, 1995) on computers with a varying number of IBM AC-922 nodes.

**FIGURE 3:**
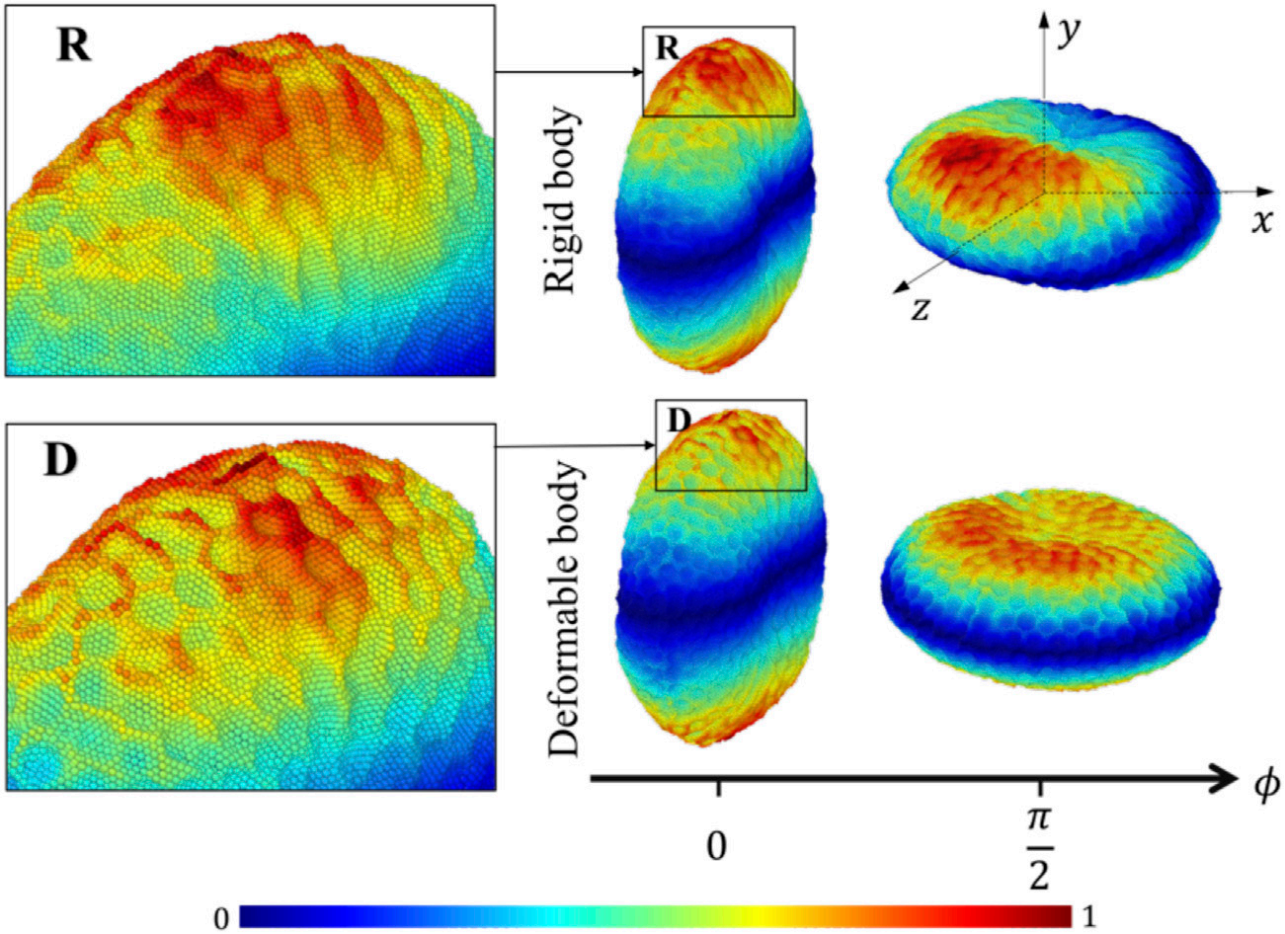
Speed distributions for representative rigid and deformable platelets at 100 dyne/cm^2^.

The platelet and the flow evolve according to their mutual interactions and our MSM (Zhang N. et al., 2014; Zhang et al., 2015) captures these motions at molecular details including the platelet’s speed distributions. The deformability, actuating a production of noisier streaming data as shown by the rougher platelet surface (Figure 3) than the rigid body simulations, demonstrates motions of nuanced differences from that of the rigid body at high frequencies. When applying speed averaging using 2,000 timesteps, both deformable and rigid body simulations gradually reveal consistent patterns, validating our pre-processing filters and different denoising steps (*Data Processing*) for raw sequences.

### Time Stepping Algorithms

To integrate the governing equations, time stepping algorithms use time discretization with a timestep size Δt (Zhang et al., 2015). To cover 3-4 orders of magnitude temporal disparity, we introduce a new multiple time stepping scheme to mix the dissipative particle dynamics and coarse-grained molecular dynamics by utilizing four different time stepping sizes (Zhang et al., 2015). These sizes are arranged as follows: the largest for the fluids; the middle one for the fluid-platelet interface; and the two smallest for the platelet structures. As in the velocity Verlet algorithm,

$$\begin{array}{cc} v\left(t+\frac{\Delta t}{2}\right)= & v\left(t\right)+\frac{\Delta t}{2m}\cdot F\left[x\left(t\right)\right]\\ x\left(t+\Delta t\right)= & x\left(t\right)+v\left(t+\frac{\Delta t}{2}\right)\\ v\left(t+\Delta t\right)= & v\left(t+\frac{\Delta t}{2}\right)+\frac{\Delta t}{2m}\cdot F\left[x\left(t+\Delta t\right)\right] \end{array}$$

The standard time stepping algorithm uses the smallest static time step size Δt to capture the details at the finest scale at a price of computing time for unnecessarily high temporal resolutions. Our multiple time stepping algorithms (Zhang et al., 2015) significantly speed up simulations by selectively modeling platelet rotation and each of its components at varying Δt . Our recent AI-enhanced adaptive time stepping algorithm (Han et al., 2020) intelligently adapts Δt ’s to underlying biophysical states, resulting in reduced computing time while bounding the numerical errors. This work was used for simulating platelet rotations for our ground truth.

### Implementation Details of BIOL

**FIGURE 4:**
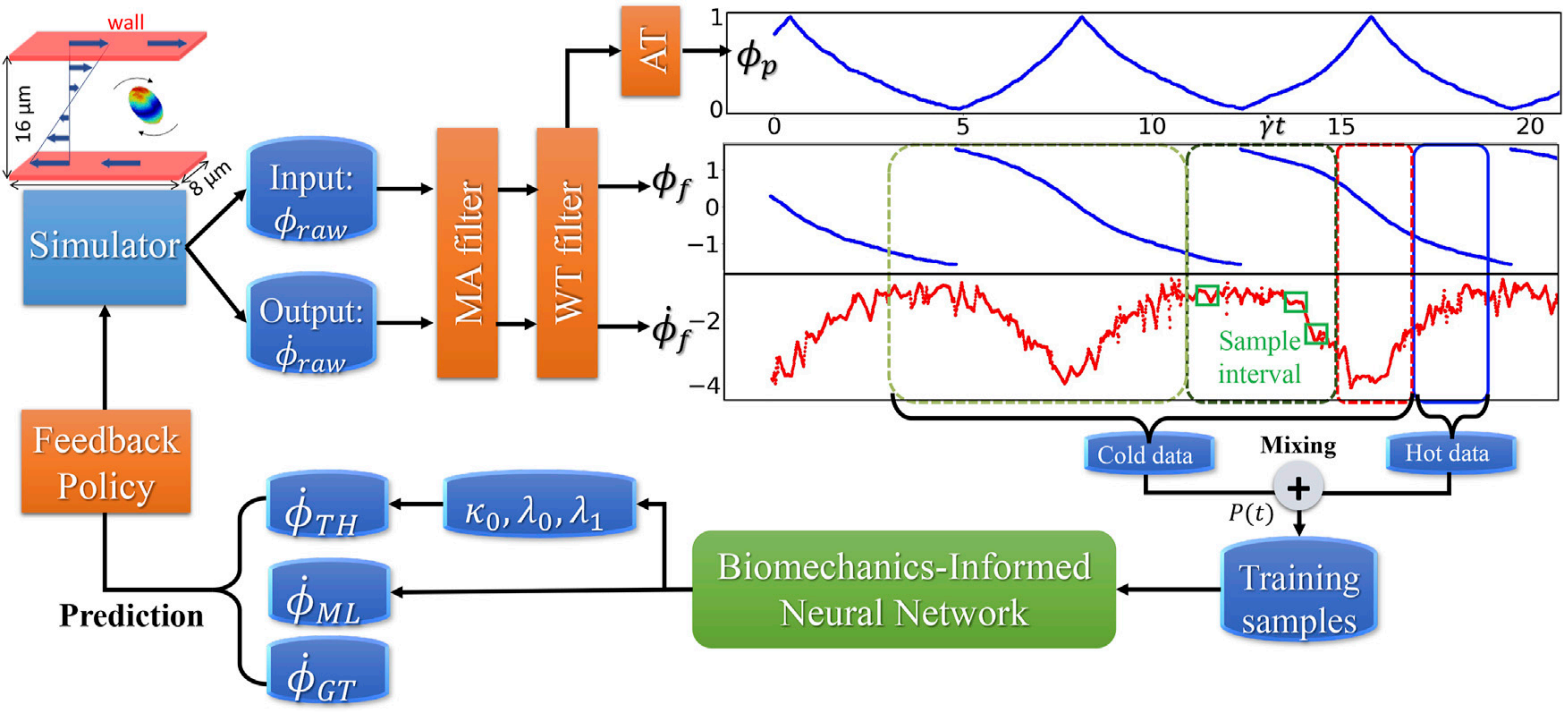
Dataflow and online learning implementations. Different colors of boxes in the data sequence plots denote online sampling windows with the total length W mixed by distribution P(t) , in which solid-line box represents hot data with length w . The green squares inside the sampling window represent sample interval with a length $N_{s}$ . All of the sampling windows will move forward with stride Δl .

We follow the dimensionless time unit in the BIOL implementation (in Figure 4), *i.e.*, the BIOL hyperparameters are pure values: the window size W=8 is chosen to be half the rotation period T/2 , and w=W/8=1 . A total 40 of time series, each of $N_{s}$ time points are sampled as the dataset for each online training process. We propose a staircase history mixing probability,

$$P\left(t\right)=\{\begin{array}{l} \frac{3}{8},\;\tau -w\leq t\leq \tau \\ \frac{1}{4},\;\tau -2w\leq t<\tau -w\\ \frac{9}{40},\;\tau -\frac{T}{2}\leq t<\tau -2w \end{array}$$

**FIGURE 5:**
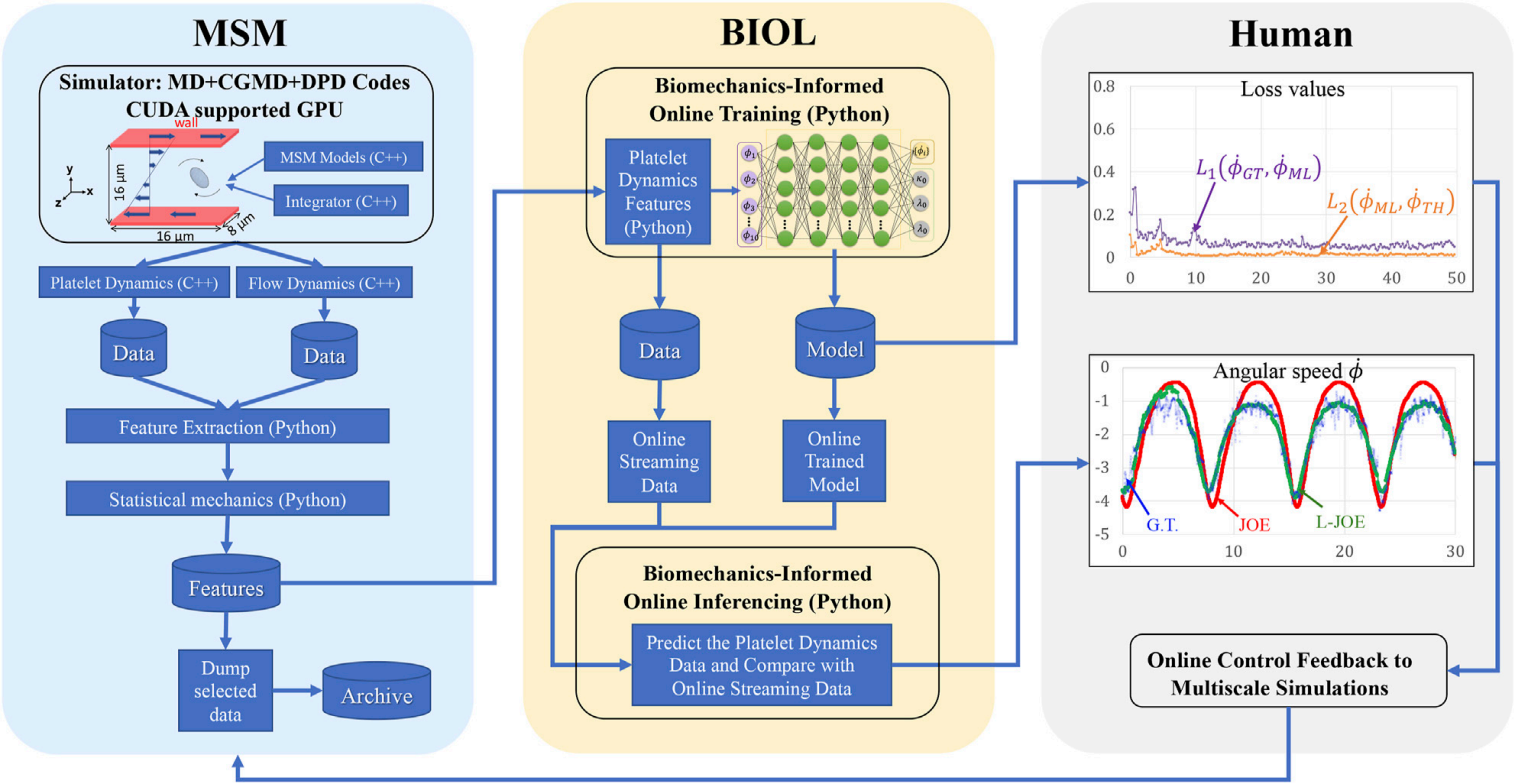
Workflow for integrated MSM and BIOL. The MSM simulator is implemented by C++ and extracted data are processed by Python. Specific features related to platelet rotation, *e.g.*, rotation angle and angular speed, are randomly sampled to feed into BIOL for solving equations of motion in time for simulation. A human, as an external intelligent side, controls the learning frequency and feedback to MSM with online predictions.

Streaming data are sampled from the most recent half period [τ−T/2,τ] with frequencies {12,10,9,9} . To denoise the high-frequency oscillation in the platelet dynamics (*Multiscale Models for Deformable Platelets*), both the raw ϕ(t) and $\dot{\phi }\left(t\right)$ are processed by the MA and WT filters. In MA, we set the moving window size to 0.08 and the stride to 0.005; in Eq. 1, we used a threshold (Donoho, 1995) $\mu =\eta \sqrt{2\cdot \ln N_{s}}$ where $N_{s}$ is the length of each time series, and $\eta =\mathrm{median}\left(\left|D_{1}\right|\right)/0.6745$ , estimated from the finest scale wavelet coefficient $D_{1}$ .

The BIOL’s $N\left(\overset{↔}{W},\overset{↔}{b}\right)$ consists of four hidden layers with 20 nodes per layer, a feed-forward fully connected NN with *tanh* as the activation function (Raissi et al., 2019). The online training is carried out in every Δl simulated time. In each training process, samples collected from MSM are fed to the NN and trained for $e_{r}\left(e_{d}\right)$ epochs with respect to the rigid (deformable) body. A decaying learning rate is scheduled starting at 0.002 with a decay rate of 0.95 and a minimum value of 0.0005. The BIOL loss function is used to train the $N\left(\overset{↔}{W},\overset{↔}{b}\right)$ and the parameters, with $\phi_{p}$ and $\phi_{f}$ respectively being the ground truth and minimized by the *Adam* optimizer (Kingma and Ba, 2015).

We studied the hyperparameter selection in our online learning by systematic analysis of the search space of the hyperparameters. Our model parameters and the methodologies in determining them serve as a working example to assess the performance of the approach. The search space is quite flat with a few subtle spots where the key hyperparameters affect the speed and accuracy of online learning predictions as well as HPC workloads. We present the studied learning stride Δl , sample interval length $N_{s}$ and training epoch number $e_{r}\left(e_{d}\right)$ and will discuss them more in *Online Training*.

### Integration of Learning and the Underlying Simulation

The holistic MSM and BIOL are implemented on an IBM Summit-like computer with 268 nodes each having two IBM POWER 9’s and associated V100 GPUs. Each POWER nine contains 20 cores at 3.15 GHz, 512 GB RAM, and a 1.6 TB SSD. All nodes connect with EDR Infiniband and a unified file system. For each of our experiments, 4–6 nodes each with six GPUs and 36 POWER nine CPUs were used for MSM and one node with one GPU was used for BIOL (200 epochs taking 5–8 s). We achieved a typical modeling speed of 1 ms simulated time for 27 h of simulated time. A full revolution of platelet rotation, requiring 2 ms, takes 54 h to simulate. In this work, we focus on the ML and HPC algorithms and the implementation at the expense of elaborations of the traditional HPC techniques such as overlapping communication with computation, double buffering, burst I/O, and scalability. The single platelet study enables a still-in-progress project of modeling more than 125-million-particle systems with 250 platelets.

The workflow (B) starts from the MSM simulator, which scales well to over 200 nodes, then the raw data is prepared with target features that are fed to BIOL for online training and inferencing. The training section is co-processed along with the simulator on the fly thus all the transferring arrows are streaming. Examining the ground truth and prediction, the inferencing section assesses the losses and evaluation metrics to regulate the simulator for acquiring more data, until the loss is adequate through a time-triggered stopping criterion. In a long-time simulation, this workflow recycles with a fixed learning frequency to enable model fine-tuning as MSM continues.

All involved data and model I/O are by file systems, as shown by the dataflow (Figure 4). Our MSM generated ground truth on multi-nodes and output to a single data file. After online sampling and pre-processing, the selected MSM features ϕ and $\dot{\phi }$ become $\phi_{f},\phi_{p}$ and ${\dot{\phi }}_{f}$ that are stored in Python *NumPy* array format and they are fed to the NN. The feedback is determined by $\left\{{\dot{\phi }}_{GT},{\dot{\phi }}_{ML},{\dot{\phi }}_{TH}\right\}$ . When the mean and standard deviation of loss values are smaller than pre-defined thresholds, the BIOL system will send a termination signal to terminate the simulator. In each learning process, I/O data files take up to 6 MB and model files up to 0.15 MB. There is no extreme I/O load in this problem and the file system, having a block size of 8 MB, fits well with our learning needs and costs nearly ignorable ms processing time.

## Results Analysis

### Online Training

To parse the prediction accuracy of our online training results, as well as selecting optimal hyperparameters in BIOL, we designed systematical experiments to use different sequences as training inputs, whose trained model will be evaluated by different test intervals in the future. The metric for prediction accuracy is defined as, based on $L_{3}\left({\dot{\phi }}_{GT},{\dot{\phi }}_{TH}\right)$ from *Online Training and Inferencing*,

$$\varepsilon =1-\frac{Er\left(𝓣\right)}{Rn\left(𝓣\right)}$$ (2)

where $Er\left(𝓣\right)=\sum_{t\in 𝓣}\frac{\left|{\dot{\phi }}_{GT}\left(t\right)-{\dot{\phi }}_{TH}\left(t\right)\right|}{{\dot{\phi }}_{GT}\left(t\right)}$ , Rn(𝓣)=max(𝓣)−min(𝓣) , and 𝓣 denotes the test interval.

**FIGURE 6:**
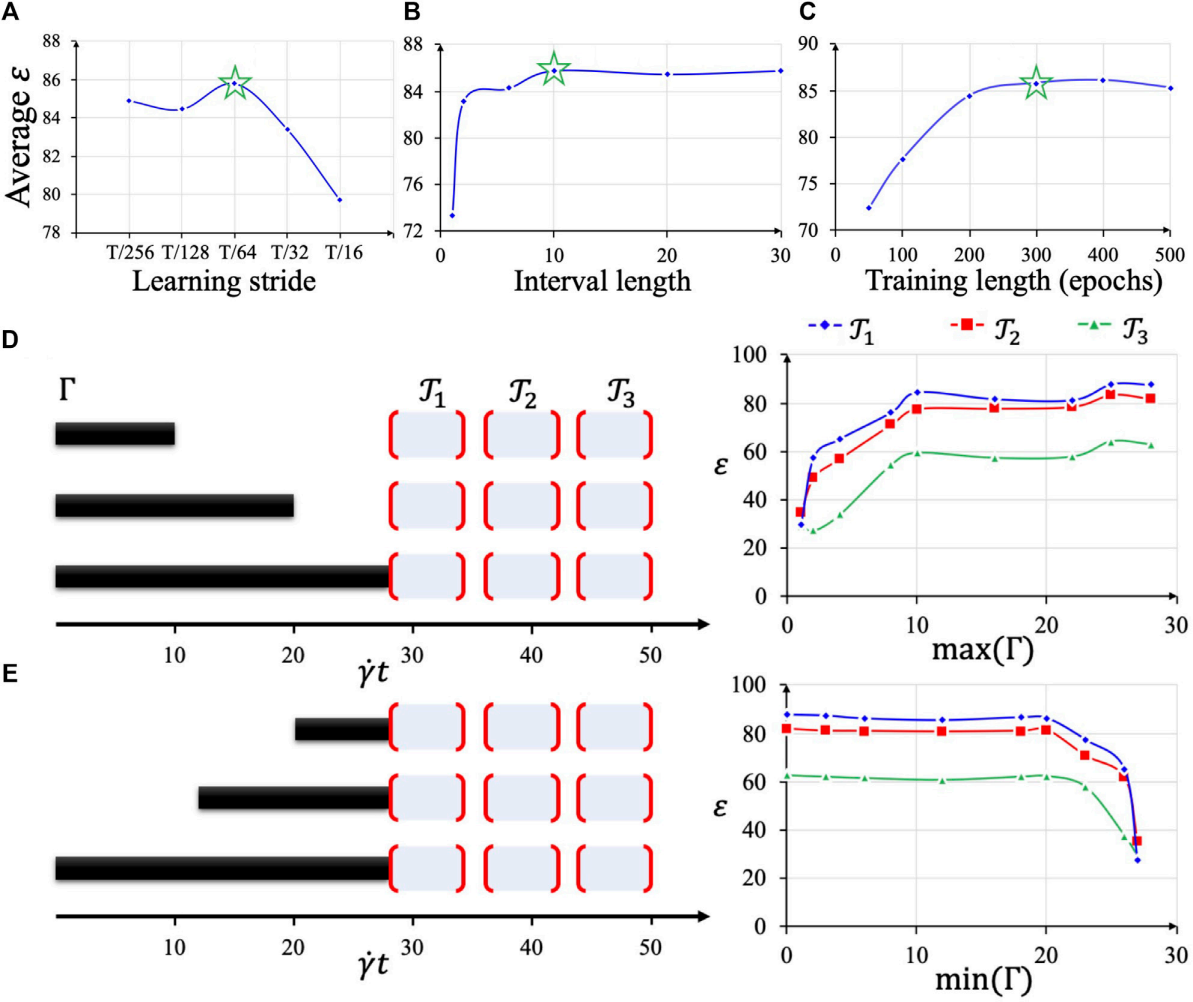
Hyperparameter selection based on average test accuracy **(A–C)**. Using the optimal hyperparameter combination, different lengths of sequences Γ **(D, E)** are fed as online training inputs, then evaluated by three different future tests ${𝓣}_{1},\;{𝓣}_{2},\;{𝓣}_{3}$ . The online predicted dynamical features are temporal sensitive. Deformable platelet at 100 dyne/cm^2^ is used as the presenting example.

For learning stride Δl , sample interval length $N_{s}$ and training epoch number $e_{r}\left(e_{d}\right)$ , we searched possible values of these hyperparameters and evaluated the average accuracy among three training and testing interval setups. Measured by dimensionless time unit, the three training intervals are [0, 16], [0,32], [0,44] and respectively the three future test intervals are [16, 24], [32, 40], [44, 50]. The experiments follow the single variable principle and the other hyperparameter values were selected in *Implementation Details of BIOL*. Δl controls the number of online learning processes since for the same length of simulation sequence, smaller Δl means more learning processes, longer training time, and simulation storage compacity. $N_{s}$ controls the length of the training element which implies the shortest continuous time series in BIOL. Larger $N_{s}$ slightly increases training time and I/O load. To avoid both underfitting and overfitting, we must select the proper training epoch number in each learning process and the balanced training time and accuracy for a trade-off. From Figure 6A–C, the optimal values selected are Δl=T/64=0.25 , $N_{s}=10,$ $e_{r}=200,\;e_{d}=300$ (rigid platelet simulations are more stable thus smaller training epoch number is selected).

After the optimal hyperparameter $\tilde{\theta }=\mathrm{argma}x_{\theta }\varepsilon$ is determined, we explore the influence of varying Γ and 𝓣 on $\varepsilon \left(\Gamma ,𝓣,\tilde{\theta }\right)$ to reveal the sensitivity and reliability of our BIOL predictions. In Figure 6D, we set the starting point of Γ to be all 0 and change the upper bounds to test the future predictions on three equally spaced ${𝓣}_{1}=\left[28,34\right],{𝓣}_{2}=\left[36,42\right],{𝓣}_{3}=\left[44,50\right]$ . For each model trained on a specific Γ , the accuracy, described in Eq. 2, decreases from near future test to late future, showing the sensitivity of BIOL. Larger max(Γ) , meaning longer training, performs a converged accuracy ε with the critical point near 8, which equals T/2 . In Figure 6E, the ending point of Γ is set to be 28, which means hot data are included in the training. The varying min(Γ) controls how far back the historical data were used in the training. A similar converged trend is observed with a critical point near 20, resulting in a max(Γ)−min(Γ)=8 . This manifests the reliability of BIOL as long as enough hot data are included in the training, the accuracy is comparable with those with longer cold data.

**FIGURE 7:**
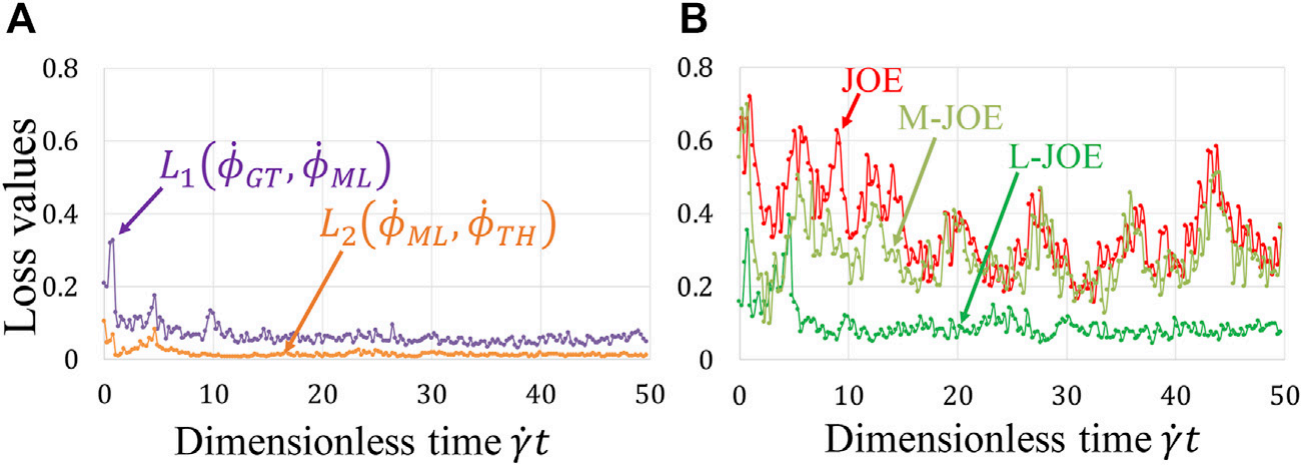
Loss function during online learning **(A)** and prediction accuracy $L_{3}\left({\dot{\phi }}_{GT},{\dot{\phi }}_{TH}\right)$ between JOE, M-JOE, and L-JOE **(B)**, for the rigid platelet at 50 dyne/cm^2^.

Our BIOL enables simultaneous learning for NN and equation parameters by minimizing the BIOL loss function (*Online Training and Inferencing*). The loss term values during the learning process are monitored and minimized as in Figure 7A. The NN parameters are initialized by the *Xavier* initialization (Glorot and Bengio, 2010) and trained by $L_{1}\left({\dot{\phi }}_{GT},{\dot{\phi }}_{ML}\right)$ . The equation parameters are initialized from the JOE that $\kappa_{0}=\lambda_{0}=\lambda_{1}=0$ and then trained by $L_{2}\left({\dot{\phi }}_{ML},{\dot{\phi }}_{TH}\right)$ . From the loss value trends, there are two stages in the learning process: the first stage is quickly getting the rotation patterns and learning the major ranges of parameters, in which the loss values drop dramatically; the second stage is dynamically tuning the parameters due to the subtle changing of platelet-flow interactions during the rotation process, in which the loss values may show small rises and falls. The first stage generally finishes within the first rotation period and the second stage could last as long as the simulation continues.

### Learned Parameters

**FIGURE 8:**
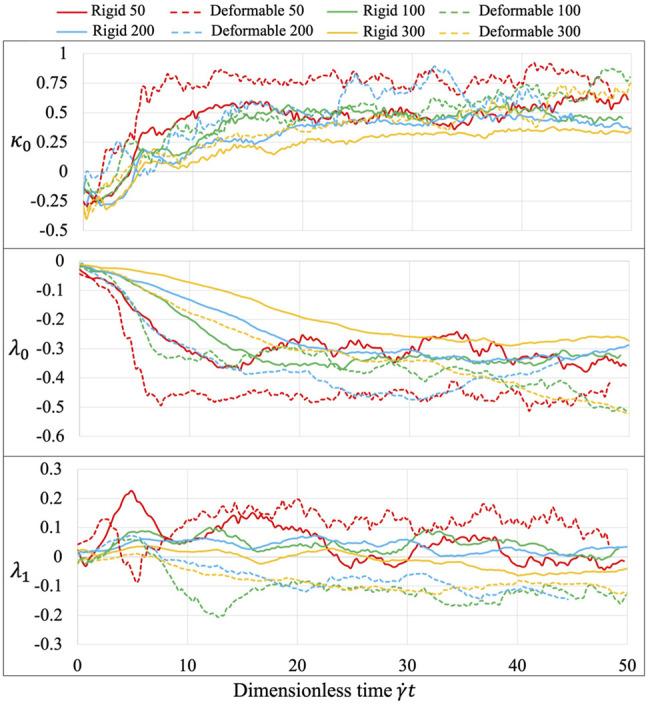
Equation parameters for different experiments. The three introduced parameters $\left\{\kappa_{0},\;\lambda_{0},\;\lambda_{1}\right\}$ are sensitive to different shear stresses and platelet body deformability.

The three learned parameters, following the 2-stage learning process (Figure 8), behave sensitively with various experimental conditions. At the second tuning stage, the fluid interaction term $\kappa_{0}$ , generally with values around 0.5, has the most significant changes in scale at this tuning stage because it reflects the platelet-flow interactions, certifying the BIOL’s reliability in detecting the sensitive dynamics of the platelet motion. Deformability manifested by $\kappa_{0}$ is also captured that the deviation from perfect oblate for which $\lambda_{0}$ converges around −0.4. The $\lambda_{0}$ , being 0 for the perfect oblate, tends to deviate from this value for deformable objects. Flow shear stress also affects $\lambda_{0}$ . For rigid body simulations, the higher shear stresses the closer $\lambda_{0}$ is to 0; the correction in cell deformability $\lambda_{1}$ converges to 0 as expected. The deformable body simulations tend to have larger absolute values of $\lambda_{1}$ .

### Analysis of Speeds

**FIGURE 9:**
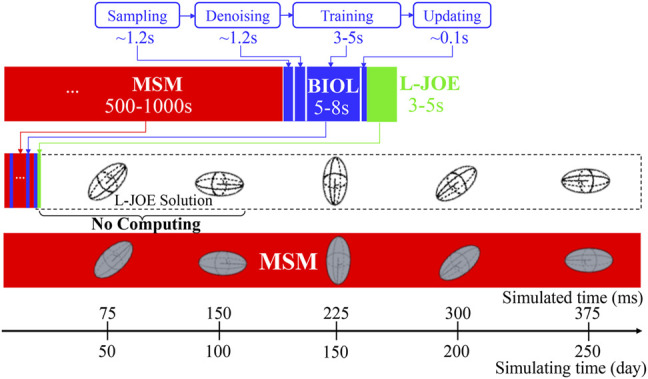
Comparison of overhead using pure MSM and our integrated methods.

**FIGURE 10:**
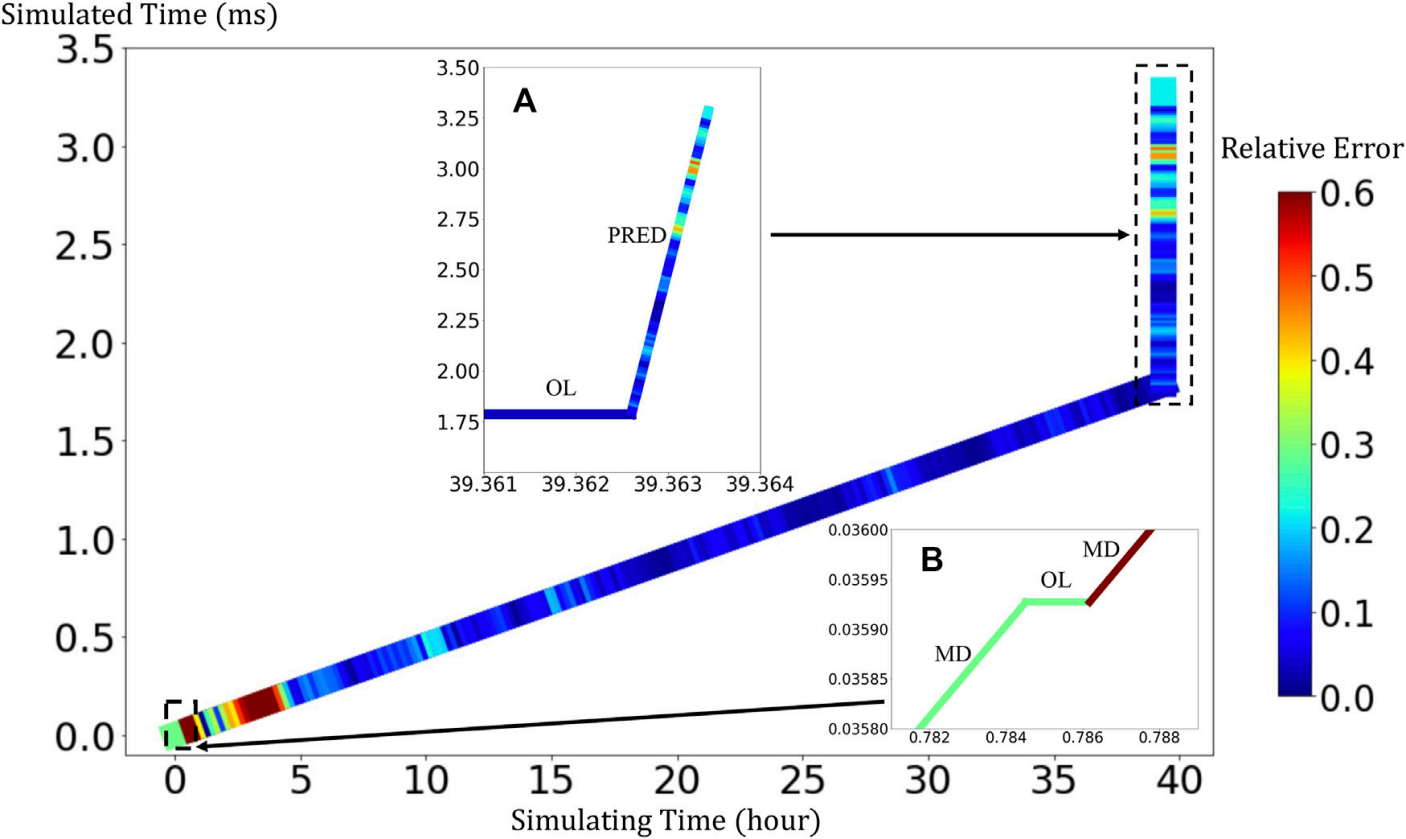
Quantitative analysis of our integrated methods. Speed is measured by simulated time over simulating time so that a larger slope means faster speed. Relative errors at different steps are expressed in a standard RGB color bar. Taking example data from rigid platelet at 300 dyne/cm^2^. MD refers to molecular dynamics, OL refers to online learning and PRED denotes predictions from BIOL. Part **(A)** and Part **(B)** are two amplified regions.

BIOL can significantly speed up the time-consuming MSM for comparable accuracy when the simulated system is sufficiently stable (Figure 9). For example, to simulate a human peripheral artery, with 5–10 mm in length, where platelets induce thrombus formation (Habib et al., 2020), conventional MSM needs 250 days to simulate a single cycle of platelet dynamics. For the same system, we use less than 2 days of MSM and negligible BIOL to achieve the same dynamics. A detailed quantitative analysis diagram (Figure 10) shows the speed and accuracy at different steps in our integrated methods. The learning round of BIOL, which is before 39.36 h simulating time from Figure 10A, may contain ∼200 learning processes before switching off MSM signaled by the online inferencing. Each learning process takes only 5–8 s as shown in Figure 10B. The prediction round of BIOL, Figure 10A, reduces unnecessary computations and replaces them with model prediction, saving ∼99% computing time. The relative error is calculated by the absolute error between predicted angular speed and ground truth values, divided by the ground truth values. Our models are at the learning stage in the first 5 h, and then maintain less than 5% relative error. To be consistent with the future prediction results in Figure 6, our model could successfully predict the angular speed with less than 10% relative error and as time goes on the error of perdition in the long future is controlled within 20%. Our more detailed accuracy analysis in the next section shows that under a stable environment, our methods are comparable to the ground truth at top scales and far more accurate than JOE solutions.

### Analysis of Accuracies

**FIGURE 11:**
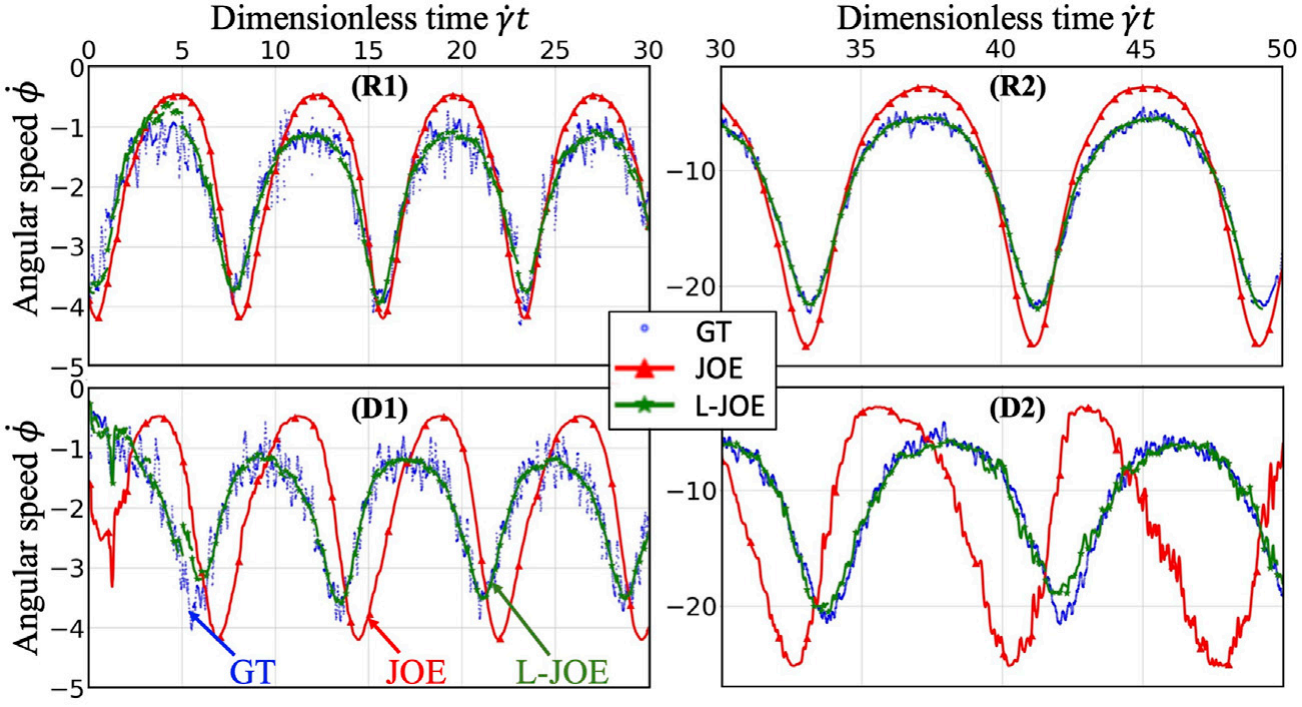
Online learning predictions of angular speed $\dot{\phi }$ for each step, taking examples from rigid and deformable platelets at 50 dyne/cm^2^ **(R1, D1)** and 300 dyne/cm^2^ **(R2, D2)**.

We performed BIOL on the total length of simulation and evaluated the overall accuracy. Compared with traditional offline learning, our online learning, while offering consistent results with offline learning, provides many advantages including determining FSI effects. For each training step, the predicted angular speed within the current moving window was plotted using the parameters learned by this step (Figure 11). The green points representing L-JOE solutions begin at JOE but gradually converge closer to the experimental data, following the 2-stage learning process observation. As expected, to an extent the longer we learn, the more accurate our predictions become. Starting with initial inaccuracies at 50 dyne/cm^2^ with the deformable case as in Figure 11D1, BIOL with L-JOE robustly learns the correct features despite numerical artifacts. For most deformable body simulations, rotation angle calculation is a difficulty since the rotation axis is instantaneous due to body shape changes. Additionally, when the simulation continues to a long timescale, we observe spectrum disorders from JOE solutions as in Figure 11D2. In these cases, JOE introduces large numerical errors, which increase as the rotation continues, while our results that are marked bold in Table 1 are far more accurate. The accuracy in Table 1 is measured by the differences between predicted angular speed and simulated ground truth, normalized by shear stress and total simulated time. By comparisons with JOE and M-JOE, our L-JOE showed smaller differences and, thus, better performance.

**TABLE 1:** Overall normalized accuracy comparisons by different physics theories.

$\frac{L_{3}\left({\dot{\phi }}_{GT},\;{\dot{\phi }}_{TH}\right)}{\dot{\gamma }t}$	Shear stress (dyne/cm^2^), rigid (R), deformable (D)							
	50		100		200		300	
	R	D	R	D	R	D	R	D
JOE	0.305	1.141	0.654	2.226	0.967	3.758	0.742	9.261
M-JOE	0.247	0.610	0.464	1.448	0.671	2.317	0.668	7.067
L-JOE	**0.081**	**0.053**	**0.085**	**0.084**	**0.138**	**0.285**	**0.302**	**0.247**
Improved Accuracy	276.5%	2052.8%	669.4%	2,518.8%	600.7%	1,218.6%	145.7%	3,649.4%

We examined the angular speeds, calculated by the JOE or predicted via BIOL using different formulas by M-JOE and L-JOE, and compared them with the MSM data via month-long HPC calculations. The time series $L_{3}\left({\dot{\phi }}_{GT},{\dot{\phi }}_{TH}\right)$ , during the online learning, shows the expected outcome that L-JOE outperforms M-JOE that, in turn, outperforms JOE as in Figure 7B. The metric for the overall accuracy is normalized as $L_{3}\left({\dot{\phi }}_{GT},\;{\dot{\phi }}_{TH}\right)/\dot{\gamma }t$ . Under all experimental conditions ( $\dot{\gamma }t\leq 50$ ) (Table 1), the L-JOE solutions are far more accurate than those of the JOE and M-JOE. The normalized accuracy metric between predictions and ground truths is calculated and the L-JOE improved up to 30 times accuracy, from JOE.

The M-JOE adds two correction terms with two independent parameters $\beta_{1}$ and $\beta_{2}$ to JOE, learnable by BIOL. To use the same first principal term, we set $\kappa_{0}=\lambda_{0}=0$ in our L-JOE and make $\lambda_{1}$ to be a learnable parameter by BIOL. Rigid body platelet at 100 dyne/cm^2^ was used as an example, the results show that the 2-parameter M-JOE has comparable accuracy with L-JOE using only one learnable parameter, reducing one degree of freedom in the parameter space. As we add parameters, L-JOE unsurprisingly gains accuracy as shown in Table 2. Two more generalized conditions were tested with $\lambda_{0},\lambda_{1}$ as learnable parameters and $\kappa_{0}=0;$ $\kappa_{0},\;\lambda_{1}$ as learnable parameters and $\lambda_{0}=0$ . As expected, the more learnable parameters we generalized, the more accurate our predictions become. The complete form of L-JOE with all three parameters $\kappa_{0},\lambda_{0},\lambda_{1}$ has the best accuracy and most stable equation parameter trends (*Learned Parameters*).

**TABLE 2:** Improvement from M-JOE to L-JOE.

Theory	M-JOE	L-JOE $\left(\lambda_{1}\right)$	L-JOE $\left(\lambda_{0},\lambda_{1}\right)$	L-JOE $\left(\kappa_{0},\lambda_{1}\right)$	L-JOE $\left(\kappa_{0},\lambda_{0},\lambda_{1}\right)$
$L_{3}\left({\dot{\phi }}_{GT},\;{\dot{\phi }}_{TH}\right)/\dot{\gamma }t$	0.464	0.385	0.319	0.288	0.085

## Discussion

In the present study, the highly celebrated century-old JOE (Jeffery, 1922), which describes the motion of a rigid ellipsoid in a steady viscous shear flow, was generalized and enabled by MSM on HPC and online ML, to analyze the motion of living cells such as the oblate-shaped human platelets in the shear blood flow. JOE demonstrated that oblates tumble with infinitely many marginally stable periodic orbits, while small perturbations of the flow conditions or the oblate characteristics may lead to substantial variations in the motion of the oblate, as evident for cells in the shear blood flow, modeled by MSM. A platelet, commonly approximated as a rigid body due to insufficient modeling resources, deviates significantly from being rigid and, thus, JOE fails badly in capturing the essentials of the motion of a true, *i.e.*, deformable, platelet. Proven by numerical experiments, our L-JOE rectifies JOE by three additional parameters, predicted by BIOL with ground truth from MSM. The reliability of L-JOE was examined by verifying it against the two-parameter M-JOE using systematic parameter analysis. L-JOE is expected to find broad applications in studying a plethora of deformable objects, including red and white blood cells, in flows, and more complex platelet dynamics like activation, adhesion, and aggregation.

Online learning provides time-dependent predictive models, avoiding burst I/O load and heavy storage compacity. The online learned models, leveraging on the use of both hot and cold data, perform better than traditional offline learning. For time-dependent cell dynamics, BIOL adaptively trains the predictive models which are sensitive to system changes. The learned model parameters may vary along with the states because of the volatility of the simulated system. Furthermore, we are exploring with a fixed learning frequency and reversible MD states, the MSM restarts, and the BIOL fine-tunes the prediction to avoid the accumulation of prediction error, until a signal of terminating MSM.

The L-JOE provides insights into the dynamics and BIOL determines the parameters. Together, they offer a fuller description. Our study of L-JOE and BIOL epitomizes the latest trends of the quick and accurate discovery of knowledgebase from data and basic science. We expect to expand L-JOE to all three dimensions for additional corrections that are subtle but our current study, under most conditions, captures the essential motion including the covering rotation at a constant rate around its major (or minor) axis, in the flow direction (Qi and Luo, 2003). For the NN in BIOL, more advanced architectures, *e.g.*, LSTM and GRU, may enhance the feed-forward DNN, a simple model working properly now.

This work conceptualized a novel architecture of coupling ML and HPC, leading to mainstream approaches for enabling HPC applications of unprecedented space and time resolutions and size as well as scientific realness, without sacrificing accuracy. It also posed new challenges and inspired new designs of next-generation supercomputer architectures involving ML. Applying the integrated L-JOE and BIOL, we analyzed the cell motion in biofluids. The platelet-specific equation and their learned parameters accurately capture the motion of platelets at a wide variety of flow conditions. This mathematics- and science-informed intelligent system enables a deeper understanding of complex biological systems and, as a bonus, may provide insights for conceptualizing a novel architecture of coupling ML and HPC.

## Data Availability

The raw data supporting the conclusions of this article will be made available by the authors, without undue reservation.

## Supplementary Material

Supplementary Video S1 Animations of the BIOL framework for deformable body platelet simulations.

Supplementary Video S2 Animations of the BIOL framework for rigid body platelet simulations.
